# The Characterization of a Novel D-allulose 3-Epimerase from *Blautia produca* and Its Application in D-allulose Production

**DOI:** 10.3390/foods11203225

**Published:** 2022-10-15

**Authors:** Xinrui Tang, Yingfeng An, Muhammad Waheed Iqbal, Hongri Cong, Guoyan Zhang, Yufei Zhang, Yuvaraj Ravikumar, Hossain M. Zabed, Mei Zhao, Haixing Zhou, Xianghui Qi

**Affiliations:** 1 School of Food and Biological Engineering, Jiangsu University, 301 Xuefu Road, Zhenjiang 212013, China; 2 College of Biosciences and Biotechnology, Shenyang Agricultural University, 120 Dongling Road, Shenyang 110161, China

**Keywords:** D-allulose 3-epimerase, D-allulose, *Blautia produca*, probiotics, biochemical characterization, biotransformation

## Abstract

D-allulose is a natural rare sugar with important physiological properties that is used in food, health care items, and even the pharmaceutical industry. In the current study, a novel D-allulose 3-epimerase gene (Bp-DAE) from the probiotic strain *Blautia produca* was discovered for the production and characterization of an enzyme known as Bp-DAE that can epimerize D-fructose into D-allulose. Bp-DAE was strictly dependent on metals (Mn^2+^ and Co^2+^), and the addition of 1 mM of Mn^2+^ could enhance the half-life of Bp-DAE at 55 °C from 60 to 180 min. It exhibited optimal activity in a pH of 8 and 55 °C, and the *K_m_* values of Bp-DAE for the different substrates D-fructose and D-allulose were 235.7 and 150.7 mM, respectively. Bp-DAE was used for the transformation from 500 g/L D-fructose to 150 g/L D-allulose and exhibited a 30% of conversion yield during biotransformation. Furthermore, it was possible to employ the food-grade microbial species *Bacillus subtilis* for the production of D-allulose using a technique of whole-cell catalysis to circumvent the laborious process of enzyme purification and to obtain a more stable biocatalyst. This method also yields a 30% conversion yield.

## 1. Introduction

Recently, different risk factors such as obesity, hypertension, and diabetes have been rising quickly around the world. The widespread occurrence of these chronic illnesses is strongly correlated with the excessive consumption of meals high in simple carbs and fat [1]. Therefore, in the field of food, nutrition, health care, and pharmacology, calorie-free and low-calorie sweetener alternatives such as rare sugars are gaining increasing attention [2]. Monosaccharides and their derivatives, which are infrequent, are referred to as rare sugars. D-allulose, commonly known as D-psicose belongs to a naturally rare sugar rather than an artificial sweetener, is one of the most significant rare sugars that has been the focus of most research. Several years ago, the US of Food and Drug Administration (FDA) identified it as generally regarded as safe (GRAS) and could be used as a component of food or food additives products [3,4]. The taste, functionality, and sweetness of D-allulose are essentially identical to those of sucrose, and it has little caloric content [5]. Furthermore, D-allulose possesses several physiological functions, such as the prevention of obesity and diabetes [6,7], treatment of hypoglycemia [8,9], metabolism of fat [10,11], and enhancement of antioxidant activity [12]. Additionally, it has great promise in the field of food, agriculture, and medicine as well as health and fitness.

Due to its rarity in nature, its large-scale application presents significant challenges and gains more attention from researchers. The previous studies elaborate that D-allulose was produced from a chemical method in the 1960s but it featured several unexpected drawbacks such as different side effects, grueling purifying procedures, and significant contaminations [13,14]. On the contrary, biosynthesis approaches such as enzymatic synthesis, provide many advantages, including high specificity, simple and convenient purification steps, and environmentally friendly features [15]. Consequently, biological production has progressively replaced other methods as the primary way to produce D-allulose [16]. It is worth our attention that in recent years, many studies have been focused on enzyme-catalyzed and microbiological methods for synthesis of D-allulose based on the Izumoring strategy [17]. The Izumoring strategy is an effective method proposed in 2006 for the bioproduction of various kinds of rare hexoses [18] and also involves D-allulose 3-epimerase (DAE), D-tagatose 3-epimerase (DTE), polyol dehydrogenases, and aldose isomerases [19]. Consequently, D-fructose can be epimerized at the C-3 position to produce D-allulose by DTEs or DAEs [20]. Due to the substrate specificity, catalytic property, and specific enzyme activity of D-tagatose epimerase and D-allulose epimerase being different enzymes and are named DTE and DAE, respectively [21,22]. However, they are collectively known as DTE family enzymes, and they are the primary enzymes played a role in the biosynthesis of D-fructose to D-allulose [16]. The DTE was discovered in 1994 from *Pseudomonas cichorii* ST-24 microbial strain [23]. According to the previous studies, there are many strains that have been discovered for the production of DTE such as *Agrobacterium tumefaciens* [21], *Rhodobacter sphaeroides* [24], *Clostridium cellulolyticum* H10 [25], *Ruminococcus* sp. [26], *Clostridium* sp. [27], *Clostridium bolteae* [28], *Dorea* sp. CAG317 [29], *Treponema primitia* ZAS-1 [30], *Flavonifractor plautii* [31], *Arthrobacter globiformis* M30 [32], *Agrobacterium sp*. ATCC 31749 [33], *Sinorhizobium sp. RSC Adv*. [34], *Novibacillus thermophilus* [35]. Table 1 goes into more detail on the catalytic properties of these DTE family enzymes. The optimal industrial conditions for D-allulose production are relatively high temperature and a neutral or slightly acidic environment; while the former can lower the substrate’s viscosity and make D-fructose easier to convert to D-allulose [36], the latter can decrease unexpected by-product formations [28]. Hence, low thermal stability is the main obstacle to the industrial production of D-allulose. Although site-directed mutagenesis as per previous studies can improve the thermal stability of DTEs [37], fewer studies exhibited that these mutations can reduce the catalytic activity of the enzyme [36]. According to one study, DTE extracted from the genes found in the macro-genome resource of the hot water exhibited high thermostability at 60 °C to 70 °C [35]. Probiotics are also sources of several DTEs, including the DTE-CM gene from *Christensenella minuta* DSM 22607 [20], which is produced in accordance with food safety regulations.

Consequently, it is necessary to excavate new enzymes that possess intrinsic properties according to the industrial demands. Additionally, the screening of sequence-based metagenomic library can be used to identify relevant DTE family enzymes [20]. Due to the availability of an abundance of sequence data, it is feasible to discover new DTEs from safe and little investigated sources and even extreme environments. In this work, one unique putative DAE (Bp-DAE) from the *Blautia produca* genome was discovered using a sequence similarity by blast tool from NCBI. The putative gene of DAE was cloned in the pANY1 vector, expressed in *Escherichia coli* BL21 (DE3), then purified. The biochemical characterization, including optimal temperature, pH, metal ion, thermostability, molecular mass, and general properties such as substrate specificity and kinetic parameters, was investigated. By conducting molecular docking with different substrates closely linked residues were identified. Furthermore, considering the safety of the strain [38], we used the *Bacillus subtilis* as the host to express Bp-DAE and the recombinant *Bacillus subtilis* cells were employed for the biosynthesis of D-allulose from D-fructose. The experimental results elaborate that Bp-DAE could transform D-fructose into D-allulose which could be used to reduce the cost of downstream industries.

## 2. Materials and Methods

### 2.1. Strains, Media, and Materials

The pANY1 vector was presented by the Shenyang Agricultural University, China. *Escherichia coli* DH5α and the host strain, *Escherichia coli* BL21 (DE3), were purchased from Stratagene (La Jolla, CA, USA). The shuttle vector, pP43NMK, and the host strain, *Bacillus subtilis* WB800N, were purchased from Fenghui Biotechnology Co., Ltd., (Hunan, China).

The cloning host, *Escherichia coli* DH5α, was grown at 37 °C and 220 rpm for 12 h in Luria-Bertani (LB) medium supplemented with Kanamycin (50 μg/mL). And the expression host strain, *Escherichia coli* BL21 (DE3), was cultivated in LB medium containing 50 μg/mL Kanamycin at 37 °C for 3~4 h and then supplemented with IPTG (1 mmol/mL) at 25 °C for 12 h. *Bacillus subtilis* WB800N was grown at 37 °C and 220 rpm for 24 h in Terrific Broth (TB) medium containing 50 μg/mL erythromycin.

The Ni^2+^-NTA resin was procured from Sangon Biotech Co., Ltd. (Shanghai, China). The main chemical reagents such as D-allulose, D-fructose, erythromycin, kanamycin, and isopropyl *β*-D-1-thiogalactopyranoside (IPTG), etc. were purchased from TCI (Tokyo, Japan), Aladdin (Shanghai, China), Macklin (Shanghai, China) and the Sinopharm Chemical Reagent (Beijing, China). Plasmid extraction and gene purification kits, the PCR required high fidelity enzymes, restriction endonuclease, and protein marker etc. were purchased from Vazyme (Nanjing, China). All of these chemicals involved in the reaction were analytical grade unless otherwise specified.

### 2.2. Gene Mining and Phylogenetic Analysis

According to the reported DTEs, *Pseudomonas cichorii* ST-24 DTE (Genebank ID: BAA24429.1) was used as the probe during the selection of novel DTEs in the NCBI database using the Blast tool. We obtained the potential DAE protein sequence originating from *Blautia produca* (NCBI accession number: WP_148391986.1). The phylogenetic tree of the different DTEs and DAEs was constructed by MEGA 11. The multiple sequences alignment of Bp-DAE was performed by ClustalX and ESPript.

### 2.3. Gene Cloning and Expression

The gene encoding Bp-DAE was ligated with 6×His-tag and optimized according to the *E. coli* codon preference, chemically synthesized as well as inserted between the *Pst* I and *BamH* I endonuclease sites of the expression vector pANY1. The recombinant plasmid, named as pANY1-*Bp-dae* and was then transformed into *E. coli* BL21 (DE3) cells in order to check the expression and its expression level.

The above-mentioned *E. coli* strains were cultivated in 0.3 L LB medium supplemented with 50 μg/mL Kanamycin at 37 °C, 220 rpm. Add 1 mmol/mL IPTG into the medium as soon as the OD_600_ reached 0.6~0.8, which takes about 3 to 4 h, and then the temperature was shifted to 25 °C, the shaking speed was shifted to 150 rpm, the induction was about to start and would last 12 h. After the induction phase, the harvested cultures were collected by centrifugation at 6000 rpm for 10 min at 4 °C. Then the cells collected by centrifugation were washed twice with 0.9% NaCl solution, and about 0.06 g of cells were resuspended in 15 mL of 50 mM Na_2_HPO_4_/NaH_2_PO_4_ buffer (pH 7.0), ultrasonic crushing of it on ice for 15 min (500 W, 3 s working, 3 s stopping), centrifuged to collect the crude cell extract, and then filtered through 0.22 μm MCE filter membrane to obtain the crude enzyme that used for the next study and subsequent enzyme purification.

### 2.4. Purification of Enzyme and SDS-PAGE Analysis

First, loading 5 mL binding buffer (pH 7.0) that contained 50 mM Na_2_HPO_4_/NaH_2_PO_4_ and 150 mM NaCl into the Chelating Sepharose Fast Flow resin column (1.0 cm × 10.0 cm) that contained Ni^2+^ for equilibrating with the column. Then, loading the obtained crude enzyme into the column twice until the target protein is bound to the Ni^2+^ column as much as possible. The unbound proteins were eluted away with 5 mL washing buffer (pH 7.0) that contained 50 mM Na_2_HPO_4_/NaH_2_PO_4_, 150 mM NaCl and 50 mM imidazole. Last, the bound protein BP-DAE was eluted by 5 mL elution buffer (pH 7.0) that contained 50 mM Na_2_HPO_4_/NaH_2_PO_4_, 150 mM NaCl and 500 mM imidazole. Then, 1.5 mL centrifuge tube was used to receive the eluate and changed to a new tube for every full 1 mL until finished. In general, the second and third tubes have the highest protein content. The protein purity was determined by a method, sodium dodecyl sulfate polyacrylamide gel electrophoresis (SDS-PAGE) analysis, as well as the determination of protein concentration was done by the Bradford method [39]. Thereafter, characterization experiments as well as determination of its kinetic and catalytic properties were performed with pure enzymes.

### 2.5. Enzyme Assay

The enzymatic reaction was conducted in 50 mM Na_2_HPO_4_/NaH_2_PO_4_ buffer (pH 8.0), supplemented with 50 g/L D-fructose, a certain amount of enzyme or recombinant whole-cell and 1 mM Mn^2+^ in a final volume of 1 mL at 55 °C for 10 min. Placing the sample in boiling water (100 °C) for 10 min to terminate the reaction. Then the reaction solution was centrifuged at 12,000× *g* rpm for 10 min, the supernatant was filtered and taken for analysis by high performance liquid chromatography (HPLC). Where one unit (U) of enzyme activity was defined as the amount of enzyme or recombinant cells required to generate 1 μ mol D-allulose per minute at pH 8.0 and 55 °C [35].

### 2.6. Characteristics of Enzyme

Determining the influence of pH on enzyme activity entailed using different ranges of pH (pH 5 to 11) in different buffer systems, for instance, 50 mM sodium acetate (pH 5, and 5.5), 50 mM phosphate (pH 6.0 to 8.5), and 50 mM glycine-NaOH (pH 9, 10, and 11.0) buffers. The optimum performing enzyme activity was defined as 100%, the pH stability was determined through incubating the enzyme in the above combination of pH buffers at 4 °C for 12 h, with standard enzyme activity assays, and the initial activity was assumed to be 100% at each pH.

Similarly, the effect of temperature was measured using different temperatures from 40 to 80 °C in 50 mM Na_2_HPO_4_/NaH_2_PO_4_ buffer (pH 8.0), where the best performing enzyme activity was defined as 100%; the temperature stability of the enzyme was examined through incubating the enzyme in 50 mM Na_2_HPO_4_/NaH_2_PO_4_ buffer (pH 8.0) at 30 °C, 40 °C, 55 °C, 55 °C accompanied with Mn^2+^, and 60 °C for several hours followed by standard enzyme activity assays. An unincubated sample was used as the control and its activity was defined as 100%.

The effects were investigated of the presence of different metal ions (1 mM Co^2+^, Mn^2+^, Mg^2+^, Fe^2+^, Ni^2+^, Zn^2+^, Ca^2+,^ and Cu^2+^) on the catalytic activity under optimal pH and temperature conditions. Furthermore, 10 mM EDTA was used to chelate metal ions to confirm whether metal ions are required for the catalytic reaction. A sample without added metal ions served as control, and its activity was defined as 100%.

### 2.7. Substrate Specificity and Kinetic Properties of Enzyme

The substrate specificity of Bp-DAE was determined, with standard enzyme activity assays, using a 300 mM concentration of different substrates such as D-fructose, D-allulose, D-tagatose, and D-sorbose; the optimum enzyme activity was defined as 100%.

The kinetic parameters of Bp-DAE were determined by measuring the activity using different range of substrate concentration (5 to 1500 mM), including D-fructose, D-allulose, D-tagatose, and D-sorbose. Kinetic parameters (such as *K_m_*, *k_cat_* and *V_max_*) were calculated by fitting with Michaelis–Menten plot and the corresponding Lineweaver-Burk plot using GraphPad Prism software (Version 6.0, San Diego, CA, United States).

### 2.8. Protein Homology Modeling and Substrate Molecular Docking

The homology model of Bp-DAE was auto-created in accordance with the reported DAE crystal structure using the SWISS-MODEL server. https://swissmodel.expasy.org/ (accessed on 27 May 2022). The substrate molecules, D-fructose and D-allulose, were used as the ligand to dock the enzyme Bp-DAE with Auto Dock software to perform molecular docking. The PyMOL software was used for the 3-dimensional structural model visualization of the enzyme.

### 2.9. Enzymatic Production of D-allulose

Use of the purified Bp-DAE as biocatalysts for the production of D-allulose from D-fructose. The enzymatic reaction solution was performed in a total volume of 50 mL consisting of 100, 300, and 500 g/L D-fructose and 50 mg purified Bp-DAE (450 U) in 50 mM Na_2_HPO_4_/NaH_2_PO_4_ buffer (pH 8.0) supplemented with 1 mM Mn^2+^ of the metal ion. The reactions were run at 55 °C and 200 rpm for 14 h in a shaker, samples were taken every one hour and the conversion yield was determined by HPLC.

### 2.10. Transformation of Bacillus subtilis and Whole-Cell Preparation of D-allulose

Based on the synthesized gene encoding Bp-DAE, primers were designed to clone the gene and link it to shuttle plasmid pP43NMK by the SeamLess Cloning technique and named pP43NMK-*Bp-dae*. Transfer of recombinant plasmids into *Bacillus subtilis* WB800N and expression of Bp-DAE. The recombinant *Bacillus subtilis* cells were cultured at 37 °C and 220 rpm for 24 h in Terrific Broth (TB) medium contained 50 μg/mL erythromycin. Firstly, 2 mL of cultured bacterial solution were taken and measured its OD_600_ and calculated the weight of the bacterial cells by fitting the curve of OD_600_ to the cell dry weight. The remaining bacterial cells were collected by centrifugation at 6000 rpm for 10 min at 4 °C. Then the centrifuged collected cells were washed twice with 0.9% NaCl solution. Finally, resuspend the cells with the appropriate amount of 50 mM Na_2_HPO_4_/NaH_2_PO_4_ buffer (pH 8.0) depending on the weight of the cells required for the reaction system and stored them at 4 °C for further use. As with the enzymatic method, the whole-cell catalytic reaction was also carried out in phosphate buffer (50 mM Na_2_HPO_4_/NaH_2_PO_4_ buffer, pH 8.0) containing 500 g/L D-fructose, 20 g/L recombinant *Bacillus subtilis* cells, and 1 mM Mn^2+^ in a shake flask with the final volume of 50 mL at 55 °C and 200 rpm.

### 2.11. Analysis of Products

The products involved in the reaction such as D-allulose, D-fructose, D-tagatose, and D-sorbose were analyzed by HPLC equipped with a refractive index detector (RID-20A) (Shimadzu, Japan) [40]. More details were in the filtration of the sample with a 0.22 μm MCE filter membrane prior to the sample analysis and the HPLC system was analyzed through a Ca^2+^-carbohydrate column (Hi-Plex-Ca, Agilent, Church Stretton, Shrops, UK) with its temperature of 84 °C, with deionized water as the mobile phase, and a flow rate of 0.6 mL/min.

And each experiment was repeated three times and the date were analyzed and processed using GraphPad Prism software and presented as mean value ± standard deviation (SD).

## 3. Results and Discussion

### 3.1. Screening DAEs and Sequence Analysis of Bp-DAE

The industrial use of D-allulose enzymes did much to pave the way for the mining of novel DTE family enzymes. NCBI database was the unique source for discovering potential contenders of DTE family enzymes. According to the database, a few other enzymes such as transaminase [41], glucose isomerase [42], and dehydrogenase [43] are workable. From the required information of the NCBI database, the putative protein (protein ID: WP_148391986.1) was obtained for the extraction of DAE from *Blautia produca* with 298 amino acids and 897 base pairs of nucleotides having a theoretical molecular mass of 33.7 kDa. The putative DAE, named Bp-DAE had 41.87% sequence similarity to the *Pseudomonas cichorii* DTE [23]. It was interesting to note that when Blast was run using Bp-DAE protein as a probe, the sequence shared up to 60% similarity with a *Lachnospiraceae* DAE [44]. The blast results raised the probability that Bp-DAE belongs to the DAE family enzymes and the safety of the source of strain was not ignorable during the screening process. The human gut bacteria *Blautia produca* isolated from feces whose species belonging to the *Lachnospiraceae* family had rapidly attracted more attention for its capabilities to alleviate inflammatory, metabolic disorders and its antibacterial properties against particular microorganisms since its discovery [44,45]. It could be used as a probiotic in food, medicinal and culinary preparations [46].

**Figure 1:**
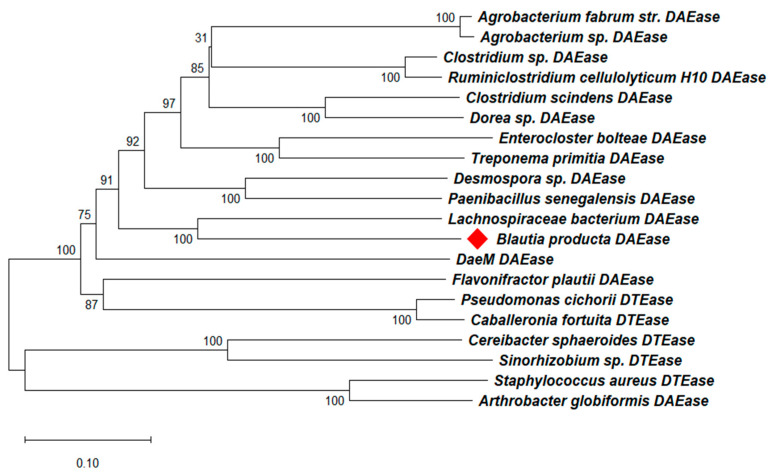
Phylogenetic analysis of Bp-DAE with known DAEs and DTEs. The red rhombus is the enzyme (Bp-DAE) used in this study.

**Figure 2:**
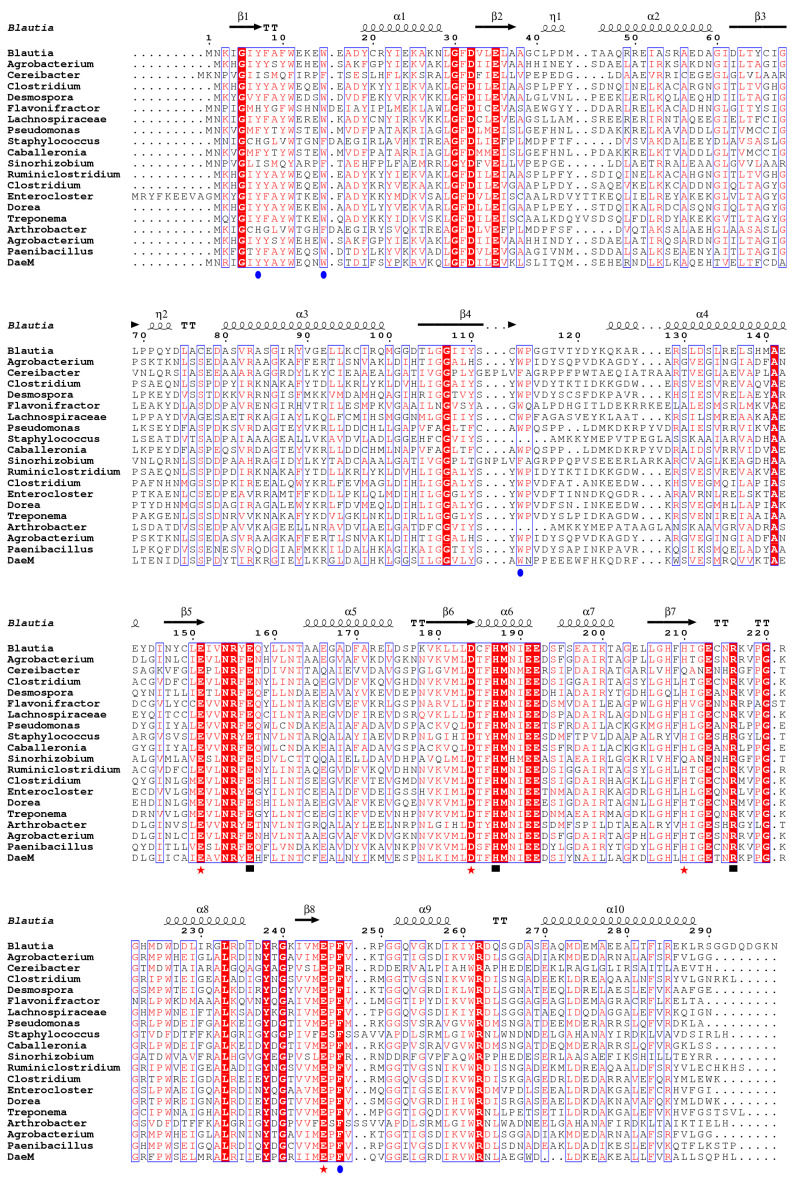
Amino acid multiple sequence alignment of Bp-DAE with known DAEs and DTEs. Sequence alignment results were performed using the software of Omega, ClustalX, and ESPript. The black wavy lines and arrows represented *α*-helix and *β*-strand, respectively. In all the sequences, amino acid residues that are identical are highlighted in red and those that are highly conserved and similar are framed in blue; the residues involved in the metal ligand sites, catalysis sites, and hydrophobic pocket forming were symbolized as red asterisks, blue dots, and black squares, respectively.

To further evaluate whether Bp-DAE is a member of the DAE family enzymes, a phylogenetic tree was formed with it using known DTE and DAE protein sequences derived from different strains. The protein sequence-based phylogenetic analysis revealed that the Bp-DAE gene had a close relationship with the previously reported *Lachnospiraceae* DAE (Figure 1). The multiple sequence alignment of Bp-DAE with the earlier reported DTE and DAE protein sequences is shown in Figure 2. After comparison, the sequence similarities were all above 60%. It exhibited that the catalytic residues including Glu151, Asp184, His210, and Glu245 are responsible for metal binding sites. The glutamate residues are also liable for the isomerization at the carbon position 3 of D-fructose. Some residues such as Glu157, Arg216, His 187, and Glu 245 were taking part in the substrate binding mechanism while Tyr7, Trp15, Trp113, and Phe247 residues provide a hydrophobic environment in the surroundings. These results were based on the comparison with the already discovered crystal structures of *Pseudomonas cichorii* DTE [47] and *Agrobacterium tumefaciens* DAE [48]. All of these results elaborate that Bp-DAE was a member of the DAE family enzymes and ability to epimerize D-fructose to D-allulose.

### 3.2. Heterologous Expression and Purification of Bp-DAE

**Figure 3:**
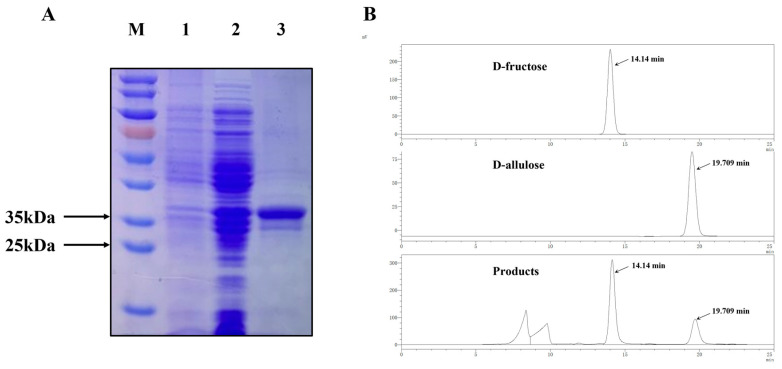
(**A**) SDS-PAGE analysis of the BP-DAE expressed in *E. coli* BL21(DE3). Lane M, standard protein marker; Lane 1, *E. coli* BL21(DE3)/pANY1 without the Bp-dae gene; Lane 2, *E. coli* BL21(DE3)/pANY1-*Bp-dae* with IPTG induction; Lane 3, purified Bp-DAE. (**B**) HPLC analysis image of the standard D-fructose, D-allulose and the biotransformation of sample in reaction. The peak diagrams in order from top to bottom are D-fructose, D-allulose, and Bp-DAE with D-fructose and D-allulose Mixed reaction solution.

The recombinant plasmid pANY1-*Bp-dae* was expressed into the host *E. coli* BL21 (DE3) as well as cultured overnight on the 50 µg/mL of kanamycin LB agar plates. A positive transformant was then selected for extended cultivation and successful induction of expression. Then the cells of recombinant bacterium *E. coli* BL21 (DE3)/pANY1-*Bp-dae* were obtained, washed, disrupt with ultrasonication, centrifuged to obtain the crude enzyme, and purified. A single and clear protein band of about 34.5 kDa was obtained by SDS-PAGE (Figure 3A). In the purified protein, a 6× His-tag tag was ligated with the encoded gene, which possessed a molecular mass of about 0.8 kDa. Therefore, the molecular mass of the purified protein was obtained as 33.7 kDa plus 0.8 kDa by SDS-PAGE analysis which become approximately 34.5 kDa and was consistent with the predicted molecular mass of Bp-DAE. Then the purified enzyme will be used for subsequent enzyme characterization. In addition, HPLC was used for the detection of the conversion from D-fructose to D-allulose to confirm the enzymatic functionality. Figure 3B shows the chromatogram of standard sugars D-fructose and D-allulose displaying 14 and 19.7 min of retention time respectively, and the reaction is also showing the same peaks on the same retention time confirming the capability of Bp-DAE to convert D-fructose to D-allulose.

### 3.3. Characteristics of Bp-DAE

**Figure 4:**
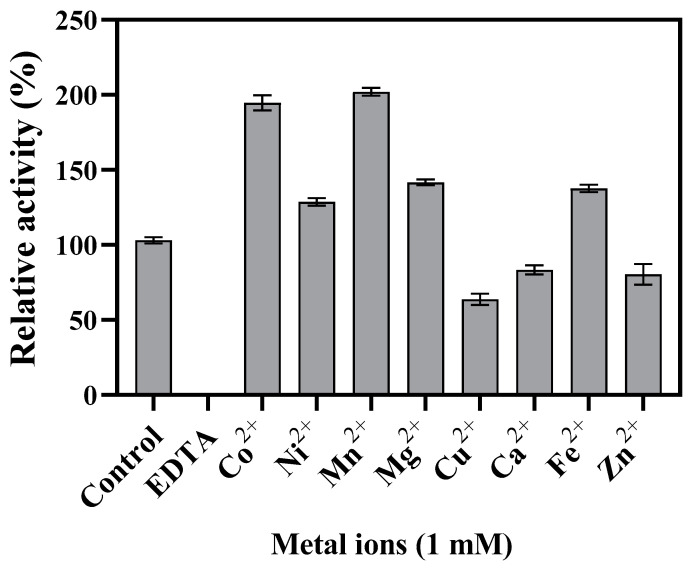
Influence of the presence of different metal ions on the enzyme activity of Bp-DAE.

The influence of the presence of various metal ions on the catalytic activity of Bp-DAE presented by Figure 4. The results exhibited that Bp-DAE possessed the maximum epimerization of 202% and 195% with 1 mM Mn^2+^ and Co^2+^ respectively. Additionally, the presence of 1 mM Mg^2+^, Fe^2+^, and Ni^2+^ slightly improved the activity of Bp-DAE but Ca^2+^, Zn^2+^, and Cu^2+^ inhibited the enzyme activity. The addition of EDTA (10 mM) entirely reduced enzyme activity, which is interesting because Bp-DAE did not exhibit any activity in the absence of the metal cofactor, demonstrating that the enzyme was strictly a metal-dependent enzyme. The present results were similar to the previously characterized DTE family enzymes such as DaeM [35] and *C. minuta* DTE [20]. However, some members of DTEs or DAEs family enzyme were not strictly metal-dependent, e.g., *Agrobacterium tumefaciens* DAE [21], *Pseudomonas cichorii* DTE [23], and *Sinorhizobium* DTE [34], but the presence of Mn^2+^ and Co^2+^ significantly enhanced their enzyme activity. The presence of Mn^2+^ and Co^2+^ nearly always boosted enzyme activity, while Cu^2+^ and Zn^2+^ almost invariably decreased the enzyme activity of DTEs and DAEs, as shown in Table 1.

**Figure 5:**
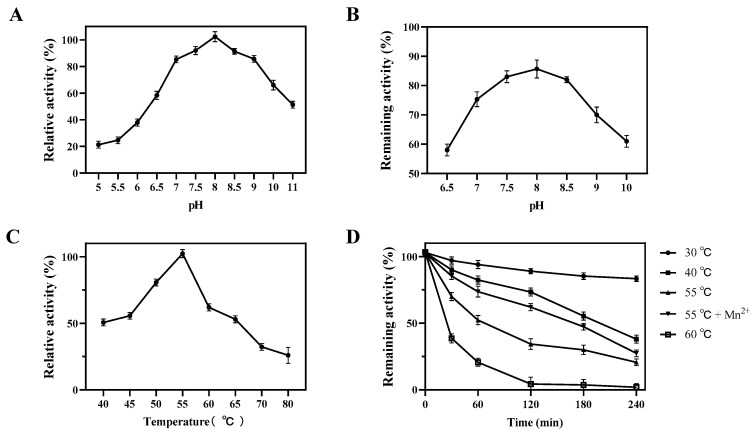
Effect of pH and temperature on the activity of Bp-DAE and its stability. (**A**) Effects of pH (**B**) pH stability; (**C**) Effect of temperature; (**D**) Thermal stability.

The effect of pH on the enzyme activity and its stability of Bp-DAE was determined at optimum temperature and different ranges of pH (5 to 11) were applied as shown in Figure 5A. Generally, a neutral to alkaline pH environment is more suitable for Bp-DAE activity. It was at optimal activity in 50 mM phosphate buffer (pH 8.0) showing 100% relative activity and maintaining above 80% of its relative activity at pH (7 to 11), suggesting that the enzyme might function in a relatively wide range of pH environments. However, the relative enzyme activity was decreased to 20% while the reaction was carried out in an acidic environment (pH 5 and 5.5) (Figure 5A). According to Table 1, it could be concluded that DTEs and DAEs generally exhibited the highest activity under neutral and weakly alkaline conditions. As shown in Figure 5B, above 60% of activity was remained at pH 6.5 to 10 after 12 h of incubation at 4 °C. This result indicated that Bp-DAE could be more stable in neutral to alkaline pH conditions.

The effect of different ranges of temperature on the enzyme activity of Bp-DAE was performed under optimum pH conditions and the results are sown in Figure 5C. The results elaborate that the highest Bp-DAE activity was shown at 55 °C, while at 50 °C, the enzyme possessed 80% of its relative activity. Bp-DAE abruptly decreased the activity with gradual increases in temperature; as shown in Figure 5C, at 70 and 80 °C the relative activity decreased to 35%. The temperature stability of Bp-DAE was measured through incubating the enzyme at different ranges of temperature (30 to 60 °C) for a period of time and the variation of activity was examined as shown in Figure 5D.

For the thermal stability assay, Bp-DAE was incubated at different temperatures (from 30 to 60 °C) for 0, 30, 60, 120, 180, and 240 min, and the variation in activity was measured (Figure 5D). The results revealed that Bp-DAE showed more than 80% of relative activity at 30 °C after 4 h and sustained 75% activity for 2 h at 40 °C while decrease to 35% after 4 h. The fluctuation in thermal stability at 55 °C was comparable with that at 40 °C, but intriguingly, with the addition of an extra 1 mM Mn^2+^, the enzyme activity was enhanced by around 35%, and the half-life was increased from 60 to 180 min compared with 55 °C. This was not an anomaly, however, since the addition of 2 mM Co^2+^ at 50 °C, increased the half-life of DaeM [49] from 297.3 to 539.2 min. Similarly, with the addition of 0.1 mM Co^2+^, the half-life of *C. cellulolyticum* DAE [25] at 60 °C increased from 10 to 408 min. Moreover, the enzyme activity of BP-DAE was almost totally inactivated after 2 hours of incubation at 60 °C. One of the key elements affecting the enzyme-catalyzed process was temperature. In the industrial production of rare sugars, higher temperatures allowed the enzymes to keep better activity because higher temperatures increased the solubility of the substrate, which sped up the enzyme reaction, thus increasing the conversion yield and to some extent reducing microbial contamination [49,50]. However, too high a temperature might trigger the Maillard reaction and lead to the thermal denaturation of the enzyme, resulting in a bad quality of sugar and low yields [49,50].

### 3.4. Substrate Specificity and Kinetic Properties of Bp-DAE

**Figure 6:**
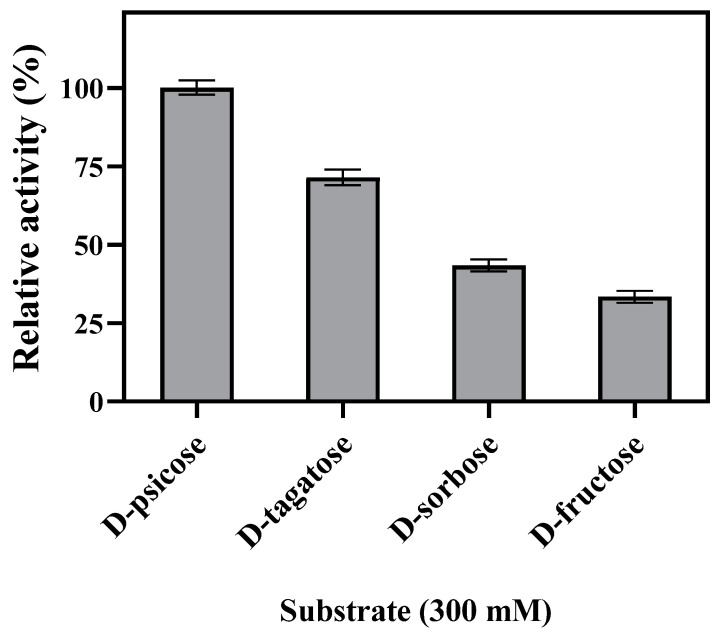
Substrate specificity of Bp-DAE.

The substrate specificity and kinetic properties of enzymes were examined to investigate the applicability of Bp-DAE in rare sugar production. Four kinds of the substrate such as D-fructose, D-allulose, D-sorbose, and D-tagatose were employed to inspect the catalytic activity of Bp-DAE and showed 38.39%, 43.39%, 71.44%, and 100% of relative activities against each substrate respectively (Figure 6). Interestingly, earlier reported DAEs and DTEs that specifically favor D-allulose or D-tagatose, similarly, Bp-DAE had also a high catalytic activity for both substrates. In the previous studies, the epimerization of DAEs or DTEs had rarely been determined against D-sorbose or its catalytic activity was very low while in the current study Bp-DAE had a high relative activity on D-sorbose compared to earlier reported DAEs or DTEs, which suggest a broad rare sugars specificity of this enzyme. The specific activity in descending order were D-allulose (5.27 ± 0.12 U/mg), D-tagatose (3.76 ± 0.13 U/mg), D-sorbose (2.29 ± 0.1 U/mg), and D-fructose (1.76 ± 0.099 U/mg). In this case, the epimerization efficiency of D-allulose was about 1.4 times higher than that of D-tagatose, thus, it is the high specificity of this protein for D-allulose that led to its precise designation as D-allulose 3-epimerase (DAE). Similar specificity preferences were present in *Ruminococcus* sp.DAE [26], *Clostridium* sp.DAE [27] and DaeM [35].

**Figure 7:**
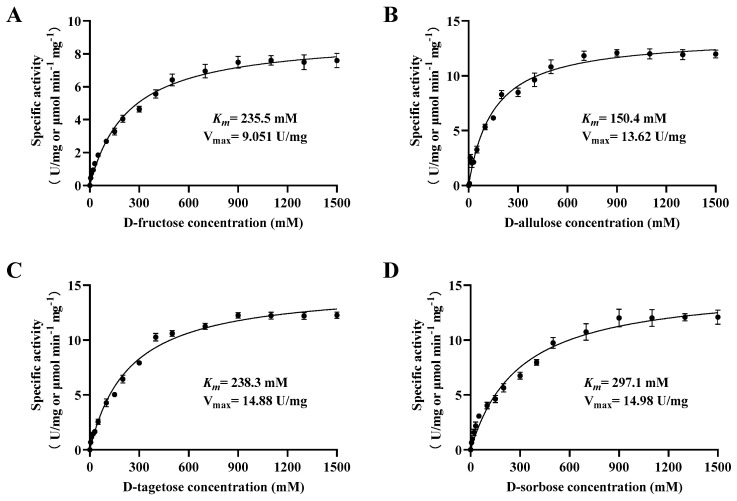
Kinetic properties of Bp-DAE. (**A**) Kinetic parameters of BP-DAE on D-fructose; (**B**) Kinetic parameters of BP-DAE on D-allulose; (**C**) Kinetic parameters of BP-DAE on D-tagatose; (**D**) Kinetic parameters of BP-DAE on D-sorbose.

The kinetic properties of Bp-DAE with different substrates such as D-fructose, D-allulose, D-tagatose, and D-sorbose according to the Lineweaver-Burk plot equation through the standard enzyme activity assay were given in Table 2 and the schematic diagram of different substrates as shown in Figure 7. The results explain that using different substrates, D-allulose showed the highest catalytic activity by exhibiting a minimum *K_m_* value (150.7 mM), the relatively high *k_cat_* value (5878 min^−1^), and the highest *k_cat_*/*K_m_* value (39.1 mM^−1^min^−1^) as compared to other substrates. The *K_m_*, *k_cat,_* and *k_cat_*/*K_m_* values of D-fructose were 235.7 mM, 3902 min^−1^, and 16.57 mM^−1^min^−1^, respectively. The Bp-DAE to D-fructose *K_m_* values from 153 to 323 mM were similar to previously reported DAEs, such as *Ruminococcus* DAE [26], *Clostridium* DAE [27], *Dorea* DAE [29], *Treponema primitia* DAE [30] and *Flavonifractor plautii* DAE [31], etc., and interestingly its *k_cat_/K_m_* value (16 mM^−1^ min^−1^) was extremely similar to that of *Ruminococcus* DAE [26]. The values of *K_m_*, *k_cat,_* and *k_cat_*/*K_m_* for D-tagatose were 238.7 mM, 6415 min^−1,^ and 26.94 mM^−1^min^−1^, respectively. While the values of *K_m_*, *k_cat_,* and *k_cat_*/*K_m_* for D-sorbose were 297.6 mM, 6457 min^−1,^ and 21.74 mM^−1^min^−1^, respectively. The kinetic properties of Bp-DAE and earlier reported DAEs and DTEs to D-allulose are presented in Table 1.

### 3.5. Structural Analysis of Bp-DAE and Enzyme-Substrate Docking

**Figure 8:**
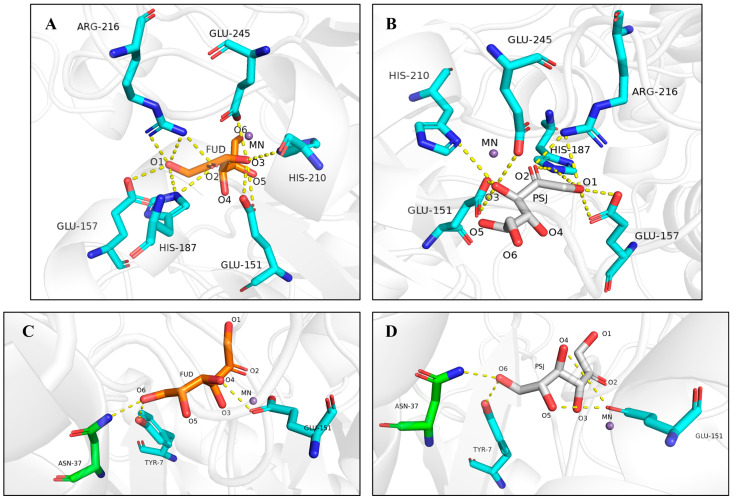
Structure diagram of Bp-DAE docking with substrate molecules. (**A**) Structure diagram of Bp-DAE docking with D-fructose; (**B**) Structure diagram of Bp-DAE docking with D-allulose. The amino acids residues in the diagram were shown as cyan sticks, and the substrate molecules of D-fructose and D-allulose were presented as orange and silvery sticks respectively, and the hydrogen bonds were shown as yellow dotted lines. Other small molecules such as Mn^2+^, was presented as a violet ball. (**C**) Hydrogen bonds were formed with the oxygen atoms at position 4, 5, and 6 of D-fructose; (**D**) Hydrogen bonds were formed with the oxygen atoms at position 4, 5, and 6 of D-allulose.

The structural homology model of Bp-DAE was created in accordance with the previously discovered DTE crystal structure of the engineered DTE PcDTE-IDF8 (SMTL ID: 4pfh.1) derived from the *Pseudomonas cichorii* DTE with some modifications. The simulated model was docked with molecules D-fructose and D-allulose respectively to investigate the mechanism of enzyme-substrate affinity. Studies had revealed that the residues around the oxygen atoms at position 1, 2, and 3 of the substrates were highly conserved and might precisely control the catalysis [51]. In the protein sequence alignment with known DAE and DTE, we knew that Bp-DAE also had similar catalytic residues and was related to substrate binding, such as Arg 216, Glu 157, His 187. The Bp-DAE-fructose structure diagram (Figure 8A) and the Bp-DAE-allulose structure diagram (Figure 8B) showed that the O1, O2, and O3 of D-fructose and D-allulose formed the same hydrogen bonds with BP-DAE protein molecule respectively including O1-Arg216, O1-Glu157, O1-His187, O2-His187, O2-Arg216, O3-Glu151, O3-His210. After molecular docking, we knew that they did combine with oxygen 1, 2, and 3 atoms of the substrate to form hydrogen bonds. Therefore, the binding-site residues at O4, O5, and O6 of the substrate were the focus of the study. According to the Bp-DAE-fructose structural analysis (Figure 8C), a total of three hydrogen bonds were established with the O4, O5, and O6 atoms of D-fructose, particularly O4-Glu151, O6-Asn37, and O6-Try7. In contrast, the Bp-DAE-allulose structure (Figure 8D) established four hydrogen bonds of O4-Glu151, O5-Glu151, O6-Asn37, and O6-Try7. Compared with the fructose substrate, D-allulose had one more hydrogen bond. Based on the biochemical and docking analyses, D-allulose would be the best substrate for Bp-DAE. It was concluded that more hydrogen bonds may have encouraged substrate alignment and restricted substrate mobility, which might be the reason for the higher affinity of BP-DAE for D-allulose [52]. Bp-DAE contains Asn, a neutral polar residue, in place of neutral nonpolar Ala37 (the corresponding residue in the engineered DTE PcDTE-IDF8), which formed one hydrogen bond with the oxygen atom at position 6 of D-fructose. In other DAEs and DTEs, where the position was usually a neutral nonpolar residue (e.g., Leu, Ala, or Ile), followed by a neutral polar (e.g., Tyr) or positive polar (e.g., His) residue [35]. The different type of amino acid residues at this position might be the reason for the higher *K_m_* of Bp-DAE and result in lower affinity for the substrate.

### 3.6. Enzymatic Production of D-allulose

**Figure 9:**
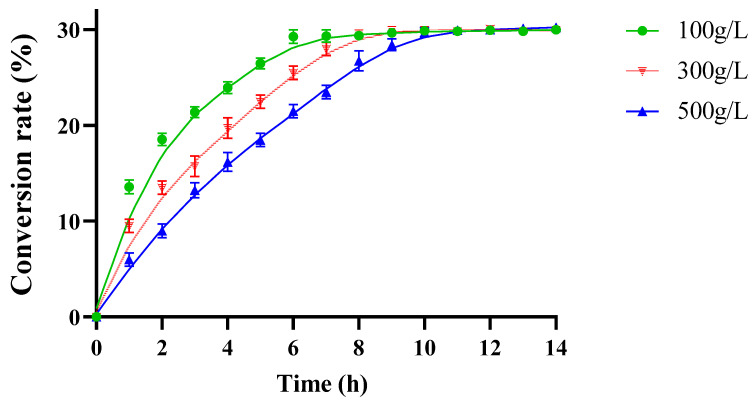
Time course of enzymatic catalysis reaction with different concentrations of D-fructose.

In a preliminary pre-experiment, 300 mM D-fructose was treated with 5 U/mL Bp-DAE under optimum reaction conditions to explore the ability of Bp-DAE to convert D-fructose to D-allulose. The highest conversion of D-allulose reached around 30% after 4.5 h of reaction. Consequently, different concentrations of substrates such as 100, 300, and 500 g/L were used with 9 U/mL of enzyme in a total volume of 50 mL under optimal conditions to further evaluate the high-level yield of D-allulose as well as the reaction equilibrium. The Bp-DAE showed 30, 90, and 150 g/L D-allulose production from 100, 300, and 500 g/L D-fructose after 6, 8, and 10 h respectively, which showed that the D-allulose/D-fructose equilibrium ratio was 30:70 (Figure 9). In the literature, the conversion yield was typically between 20 to 33% by employing different ketose 3-epimerases (as presented in Table 1). The results of the present findings elaborate that the catalytic performance of Bp-DAE with 500 g/L substrate was at a medium to a higher level but the catalytic rate of Bp-DAE was significantly lower than that of known DAEs or DTEs such as the reaction equilibrium time of *Treponema primitia* DAE with 500 g/L substrate was 6 h while for Bp-DAE was 10 h which might be due to the low binding efficiency of the substrate. Alike other enzymes for improving some features, either rational modification or random mutation was possible [53]. For example, it was feasible to improve the catalytic rate of Bp-DAE by some techniques like site-directed mutagenesis as well as random and directed evolution [36,37].

### 3.7. Whole-Cell Catalysis Production of D-allulose

**Figure 10:**
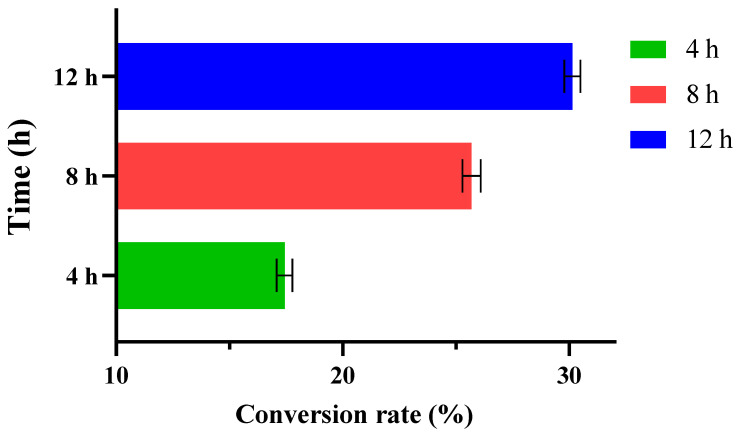
The whole-cell catalysis reaction for D-allulose production with 500 g/L D-fructose.

The whole-cell biocatalysts production has the following advantages over enzymatic production: (1) Cell culture is simple, the enzymes present in the cells have greater stability to the biocatalysts, also eliminating the need for complicated and tedious enzyme purification processes, (2) enzyme are protected by a cell wall and membranes from harsh conditions and maintain their full activity [54,55]. These advantages made the bioprocess more cost-effective and more suitable for scale up production. Therefore, the Bp-DAE gene was cloned into the pP43NMK shuttle plasmid, and *Bacillus subtilis* was used as the expression host, which was considered to be a safe-grade microbial strain. Then, 20 g/L whole cells of recombinant *Bacillus subtilis* was used with 500 g/L D-fructose substrate for a period of time under optimum enzymatic reaction conditions, and their reaction equilibrium times were recorded. The whole cell catalyst produced 17.43% of D-allulose after 4 h, 25.69% after 8 h, and reached equilibrium after 12 h with a 30.13% conversion yield by producing a yield of about 150.65 g/L (Figure 10). In contrast, the whole-cell catalytic trial with empty vector (pP43NMK shuttle plasmid without Bp-DAE) recombinant *Bacillus subtilis* cells used as control, and under a same condition, revealed that this host cell did not transform any D-fructose during the reaction. In the actual production of D-allulose, *Bacillus subtilis* was a safe-grade microbial strain and more applicable for the food industry as compared to *Escherichia coli*. Therefore, the catalysis reaction using the whole cells of *Bacillus subtilis* for D-allulose production was a desirable method [56]. In this study, *Bacillus subtilis* cells were successfully used as hosts to express Bp-DAE, which converted D-fructose to D-allulose after 12 h based on a whole-cell catalytic reaction with a conversion yield of about 30%. However, the genomic integration of Bp-DAE had an innate potentiality for higher catalytic efficiency compared with a plasmid-based transformation, which showed an instability limitation during multiple generations of plasmid vectors [56]. It is concluded that a whole-cell catalytic technique combined with the fed-batch strategy is an effective bioprocess approach to achieve a higher yield of D-allulose that could be applied to the production of many high value-added products [35].

## 4. Conclusions

In brief, we found a novel D-allulose 3-epimerase named Bp-DAE based on the NCBI database, and encoded genes were obtained from a potential probiotic *Blautia produca*. The gene source was safe and conformed to food grade production. Then the encoded genes were expressed in *E. coli* BL21 (DE3) for the extraction of Bp-DAE and purified the enzyme for characterization. The Bp-DAE exhibited neutral to alkaline pH (optimum 8.0) at optimum temperature 55 °C with good catalytic activity and broad substrate specificity. In addition, it could also maintain activity in slightly acidic environment, which was helpful for industrial production of D-allulose. The current study showed that Bp-DAE enzyme was strictly dependent on metal ions and almost inactivated in the absence of metal ions, its catalytic activity has been greatly increased with the addition of Co^2+^ and Mn^2+^ ions. Moreover, whole-cell catalysis by employing Bp-DAE-transformed *Bacillus subtilis* cells and pure enzyme catalysis were used for the conversion of D-fructose to D-allulose, which showed a 30% conversion yield which was intermediate to high. Recently, many studies had been focused on improving the specificity of D-fructose and the thermal stability of enzymes. Conclusively, this research demonstrated that Bp-DAE could be used an effective biocatalyst for the D-allulose production that can be used for industrial purpose to decrease the price of downstream industries.

## Figures and Tables

**Table 1:** Comparison of the enzymatic properties of previous DTEs and DAEs.

Source of Enzymes	Opt. pH	Opt. TEMP. (°C)	Metal Dependence	Opt. Ion	Half-Life (min)	Opt. Substrate	*K_m_*^b^ (mM)	*k_cat_*/*K_m_*^b^ (mM^−1^min^−1^)	Conversion (%)
*Pseudomonas cichorii* [23]	7.5	60	No	Co^2+^	NR	D-tagatose	NR	NR	20
*Agrobacterium tumefaciens* [21]	8.0	50	No	Mn^2+^	64 (50 °C)	D-allulose	12	198.4	33
*Rhodobacter sphaeroides* [24]	9.0	40	No	Mn^2+^	NR	D-fructose	NR	NR	23
*Clostridium cellulolyticum* [25]	8.0	55	Yes	Co^2+^	10 (60 °C)	D-allulose	17.4	186.4	32
*Ruminococcus* sp. [26]	7.5	60	No	Mn^2+^	96 (60 °C)	D-allulose	48	50.5	28
*Clostridium* sp. [27]	8.0	65	Yes	Co^2+^	15 (60 °C)	D-allulose	227.6	141.4	25
*Clostridium bolteae* [28]	7.0	55	Yes	Co^2+^	43 (55 °C)	D-allulose	27.4	106	32
*Dorea* sp. [29]	6.0	70	Yes	Co^2+^	30 (60 °C)	D-allulose	191	412	30
*Treponema primitia* [30]	8.0	70	Yes	Mn^2+^	30 (50 °C)	D-allulose	209.1	144.3	28
*Flavonifractor plautii*. [31]	7.0	65	Yes	Co^2+^	130 (60 °C)	D-allulose	162	156	NR
*Arthrobacter globiformis* M30 [32]	7.5	70	No	Co^2+^	NR	D-allulose	31	182.7	NR
*Agrobacterium* sp. [33]	7.5–8.0	55–60	Yes	Co^2+^	75 (55 °C)	D-allulose	NR	NR	30
*Sinorhizobium* sp. [34]	8.0	50	No	Mn^2+^	NR	D-tagatose	39.8	118.2	NR
*Novibacillus thermophilus* (DAEM) [35]	7.0	80	Yes	Co^2+^	49 (80 °C)	D-allulose	NR	NR	31
*Christensenellaceae.minuta* [20]	6.0	50	Yes	Ni^2+^	40 (50 °C)	D-tagatose	53.8	124	30
*Blautia produca* ^a^	8.0	55	Yes	Mn^2+^	60 (55 °C)	D-allulose	150.7	39.1	30

NR, not reported. ^a^ Bp-DAE, the enzyme used in this study. ^b^ Determined with D-allulose as substrate.

**Table 2:** Substrate specificity and kinetic properties of Bp-DAE for different substrates.

Substrates	*K_m_* (mM)	*k_cat_* (min^−1^)	*k_cat_*/*K_m_* (mM^−1^ min^−1^)	Relative Activity (%)
D-allulose	150.7 ± 9.166	5878 ± 157.3	39.1 ± 3.216	100.1 ± 2.283
D-fructose	235.7 ± 9.938	3902 ± 213.4	16.57 ± 0.8634	33.39 ± 1.88
D-tagatose	238.7 ± 13.63	6415 ± 110.1	26.94 ± 1.686	71.44 ± 2.461
D-sorbose	297.6 ± 13.68	6457 ± 284.9	21.74 ± 1.719	43.39 ± 1.898

All experiments were repeated in triplicate, and data were presented by mean value ± standard deviation.

## Data Availability

All datasets generated for this study are included in the article.
